# Analysis of onset-to-door time and its influencing factors in Chinese patients with acute ischemic stroke during the 2020 COVID-19 epidemic: a preliminary, prospective, multicenter study

**DOI:** 10.1186/s12913-024-11088-8

**Published:** 2024-05-10

**Authors:** Yuqi Liao, Wenwei Qi, Shuting Li, Xin Shi, Xiaohong Wu, Feng Chi, Runyu Xia, Limin Qin, Liming Cao, Lijie Ren

**Affiliations:** 1 School of Medicine, Shenzhen University, Shenzhen, China; 2 National Center for Cardiovascular Diseases, Chinese Academy of Medical Sciences, Peking Union Medical College, Beijing, China; 3 Fuwai Hospital, Chinese Academy of Medical Sciences and Peking Union Medical College, Beijing, China; 4 School of Statistics, Shandong Technology and Business University, Yantai, China; 5 Department of Neurology, The First Affiliated Hospital of Shenzhen University, 3002 Sungang West Road, Futian District, Shenzhen City, 518000 China; 6 Department of Neurology, Shenzhen Second People’s Hospital, Shenzhen, China; 7 Hunan Provincial Key Laboratory of the Research and Development of Novel Pharmaceutical Preparations, Changsha Medical University, Changsha, China; 8 School of Health Management, China Medical University, Shenyang, China

**Keywords:** Acute ischemic stroke, Onset-to-door time, Pre-hospital delay, Current situation, Risk factors, China

## Abstract

**Background:**

Pre-hospital delay in China is a serious issue with unclear relevant reasons, seriously impeding the adoption of appropriate measures. Herein, we analyzed the onset-to-door time (ODT) in Chinese patients with acute ischemic stroke (AIS) and its influencing factors.

**Methods:**

We prospectively recruited 3,459 patients with AIS from nine representative tertiary general hospitals in China between January and June 2022. Patients were divided into ODT ≤ 3 h and ODT > 3 h groups. Following single-factor analysis, binary logistic regression analysis was performed to evaluate the risk factors leading to pre-hospital delay.

**Results:**

In total, 763 (21.83%) patients arrived at the hospital within 3 h of onset. After adjusting for confounding factors, the risk factors for ODT were residence in rural areas (odds ratio [OR]: 1.478, 95% credibility interval [CI]: 1.024–2.146) and hospital transfer (OR: 7.479, 95% CI: 2.548–32.337). The protective factors for ODT were location of onset ≤ 20 km from the first-visit hospital (OR: 0.355, 95% CI: 0.236–0.530), transportation by emergency medical services (OR: 0.346, 95% CI: 0.216–0.555), history of atrial fibrillation (OR: 0.375, 95% CI: 0.207–0.679), moderate stroke (OR: 0.644, 95% CI: 0.462–0.901), and severe stroke (OR: 0.506, 95% CI: 0.285–0.908).

**Conclusions:**

Most patients with AIS fail to reach a hospital within the critical 3-h window. The following measures are recommended to reduce pre-hospital delays: reasonable distribution of hospitals accessible to nearby residents, minimizing interhospital transfer, paying attention to patients with mild stroke, and encouraging patients to use ambulance services. Pre-hospital delays for patients can be reduced by implementing these measures, ultimately improving the timeliness of treatment and enhancing patient prognosis. This study was carried out amid the COVID-19 pandemic, which presented challenges and constraints.

## Background

Acute ischemic stroke (AIS) is a common acute cerebrovascular disease with high disability and mortality rate [1]. Restoring vascular recanalization and improving tissue perfusion within the time window is the key to a successful treatment. Intravenous thrombolysis (IVT) within 3 h of AIS onset can effectively improve prognosis without a significantly increased risk of death [2]. Pre-hospital delay in AIS in China is common [3–9]. However, the impact of the Coronavirus Disease 2019 (COVID-19) pandemic, which began in January 2020 [10], on these delays and their influencing factors remain unclear. This lack of clarity further impedes the formulation of improvement measures.

The global burden of stroke has increased markedly over the past 20 years, especially in developing countries [11]. In China, stroke has become the leading cause of death over the past 30 years [12]. The median onset-to-door time (ODT) for AIS in China is 15 h, and only a quarter of patients reach the hospital within 3 h [13], which is obviously longer than that in developed Western nations. Compared to those in the United States, patients in China are more prone to experiencing pre-hospital delays (1318 min vs. 644 min), resulting in a lower thrombolysis rate (2.5% vs. 8.1%) [14]. In contrast, stroke burden and mortality have declined in many developed countries, largely due to improvements in stroke prevention and acute stroke care.

Many factors are associated with ODT in patients with AIS, including age, sex, residential status, educational level, medical history, transportation to the hospital, and efficiency of emergency medical services (EMS) [5, 15, 16]. Patients’ understanding and recognition of stroke symptoms are critical for shortening the ODT [17]. EMS has been shown to reduce pre-hospital delays [18]. Pre-hospital delays differ greatly between China and developed countries, owing to the differences in education, culture, socioeconomic status, medicine, and health [19].

This study aimed to analyze the current situation of ODT in Chinese patients with AIS and its influencing factors through a large-scale, multicenter study and provide evidence for government health departments to make scientific decisions so that more patients can receive timely and optimal treatment, improving prognosis.

## Methods

### Study design

This study was a multicenter, large-sample, prospective, and observational study.

### Study participants

A total of 3495 patients with AIS were recruited from nine hospitals (including Fushun Central Hospital, Wuzhou Workers’ Hospital, Huaihua First People’s Hospital, Inner Mongolia Autonomous Region People’s Hospital, the First Affiliated Hospital of Shaoyang Medical College, Xiangxi Tujia and Miao Autonomous Prefecture People’s Hospital, Affiliated Hospital of Yan’an University, Yueyang Central Hospital, and Zhuzhou Central Hospital) certified as “stroke centers” [20] by the China National Stroke Prevention Project Committee Commission from January to June 2022.

The data collection and entry personnel in all subcenters had professional knowledge of stroke and were trained by the project manager. AIS was diagnosed according to the guidelines [21], and intracranial hemorrhage was excluded using head computed tomography or magnetic resonance imaging [22]. The stroke subtype was based on the trial of Org 10,172 in acute stroke treatment (TOAST) classification of stroke [23].

#### Inclusion criteria

1. Age ≥ 18 years;
2. AIS diagnosis;
3. Stroke onset ≤ 7 days on admission;
4. provision of consent to participate in this program.

#### Exclusion criteria

1. Diagnosis of transient ischemic attack, AIS occurring in hospitals, active malignancy, iatrogenic AIS, or cerebral venous sinus thrombosis.
2. Life expectancy less than 3 months; and
3. Diagnosed with severe mental disorders, cognitive disorders, or other conditions.

The ODT was defined as the time from the onset of stroke symptoms to admission to the hospital emergency department or outpatient clinic. For patients whose onset time was uncertain (e.g., wake-up stroke), the last known asymptomatic time was taken as the onset time.

### Study variables and groups

The variables studied included sex, age, educational level, residence status, medical insurance, wake-up stroke, first symptom of AIS, distance between onset location and first-visit hospital, transfer method for patients, whether an inter-hospital transfer was performed, medical history, pre-onset modified Rankin scale (mRS) score, stroke severity (according to the National Institutes of Health Stroke Scales [NHISS] first score after onset, moderate and severe stroke have NHISS score 5–14 and NHISS score 15–42 respectively) [24], patient’s knowledge about AIS, and TOAST classification. The division of patients based on ODT is crucial for stratifying stroke care and predicting outcomes [25]. Therefore, patients with AIS were categorized into the ODT ≤ 3 h group and ODT > 3 h group accordingly.

### Definition of AIS’s initial symptoms

In this section, we outline a comprehensive understanding of various symptoms encountered in AIS cases, ranging from common manifestations, such as vomiting or unconsciousness, to more specific indicators, including diplopia or dysarthria. The initial symptoms of AIS are defined as follows [26, 27]: (1) Vomiting: Involuntary expulsion of stomach contents through the mouth or nasal cavity. (2) Unconsciousness: Lack of response to external stimuli, coma, or other non-alert states. (3) Paralysis: Complete loss of voluntary motor function, which may affect specific body parts or one side. (4) Diplopia: Simultaneous perception of two images of the same object. (5) Aphasia: Loss or impairment of the ability to express or understand language, characterized by difficulties in speaking, expressing oneself, or understanding others. (6) Dysarthria: Unclear speech or difficulty in pronouncing words due to impaired neuromuscular control. (7) Drooping of the angle of the mouth: Noticeable drooping of one side of the mouth corner, resulting in an asymmetrical facial expression. (8) Headache: Persistent pain or discomfort experienced in the head. (9) Paresthesia: Abnormal sensations felt on the skin, such as numbness, tingling, or burning, without obvious stimulation. (10) Vertigo: Sensation of spinning or movement of the surrounding environment or oneself, often accompanied by balance disorders. 11) Other symptoms included visual disturbances that are difficulty to classify within the categories mentioned above. Detailed symptom information can be provided upon entry of specific data.

### ODT calculation method

In this study, we typically documented the precise time when the patient or a witness first noticed stroke symptoms, such as sudden weakness, speech difficulties, or visual disturbances. Alternatively, when the onset time of symptoms was unclear, the following methods were used: (a) When the patient woke up with symptoms, the time before sleep when the last symptom-free period was confirmed was considered as the onset time of symptoms [28, 29]. (b) When the exact time of symptom onset cannot be determined, the time of the last confirmed symptom-free period was considered as the onset time of symptoms [28, 29]. Subsequently, the time of the patient’s arrival at the outpatient department or emergency room, specifically at the triage entrance [30], was documented. The ODT was calculated by subtracting the recorded onset time of stroke symptoms from the time of arrival at the healthcare facility’s door. For instance: (1) Unconsciousness: If the patient lost consciousness, the time when symptoms started was determined based on witness accounts or when the patient was found. This time was considered as the onset time, and then the time when the patient arrived at the hospital was recorded to calculate ODT. (2) Headaches: For localized headaches, the time when the patient or witnesses noticed the headache starting was considered the onset time. Following this, the time of arrival at the hospital was recorded to calculate ODT.

### Statistical analysis

All statistical analyses were performed using IBM SPSS Statistics (version 26.0; IBM Corp., Armonk, N.Y., USA). The measured data with normal distribution are expressed as the mean ± standard deviation, and the independent-sample *t*-test was used for between-group comparisons. When data does not follow a normal distribution, quartiles are used to describe the data. Categorical variables are presented as counts and percentages, and the differences between the two groups were analyzed using the chi-squared test. Firstly, the differences between the ODT ≤ 3 h group and the ODT > 3 h group were analyzed using single-factor analysis; subsequently, the variables with significant differences were included in the binary logistic multivariate analysis, which typically yields confidence intervals for parameter estimates and conducts multicollinearity tests on the variables within the multivariable model. All statistical tests were two-sided, and the threshold for statistical significance was set at *P* < 0.05.

## Results

### Overview of ODT in patients with AIS (Table 1)

**Table 1:** Baseline characteristics and single factor analysis of ODT in patients with acute ischemic stroke

		ODT ≤ 3 h, %	ODT>3 h, %	*P* value
**Number of cases**		763, 21.83%	2732, 78.17%	
Gender	Male	520, 68.15%	1841, 67.39%	0.690
	Female	243, 31.85%	891, 32.61%	
Age, y		67.02 ± 11.22	65.51 ± 11.58	**0.011**
Whether patients lived alone	Yes	38, 4.98%	112, 4.10%	0.289
	No	725, 95.02%	2620, 95.90%	
Residential location	City	521, 68.28%	1341, 49.08%	**0.000**
	Rural and other areas	242, 31.72%	1391, 50.92%	
Medical insurance	Yes	638, 83.62%	2315, 84.74%	0.450
	No	125, 16.38%	417, 15.26%	
Educational level	Illiterate	32, 4.19%	219, 8.02%	**0.000**
	Primary or junior high school	397, 52.03%	1462, 53.51%	
	High school or junior college	293, 38.40%	835, 30.56%	
	University or above	41.5, 38%	216, 7.91%	
**Whether patient or his family knew the stroke emergency map**				
	Yes	37, 4.85%	115, 4.21%	0.444
	No	726, 95.15%	2617, 95.79%	
**Whether patient or his family visited the WeChat official account of the stroke emergency map**				
	Yes	14, 1.83%	35, 1.28%	0.250
	No	749, 98.17%	2697, 98.72%	
Wake-up stroke	Yes	118, 15.47%	488, 17.86%	0.122
	No	645, 84.53%	2244, 82.14%	
Distance between onset location and first-visit hospital	>20 km	135, 17.69%	1161, 42.50%	**0.000**
	≤ 20 km	628, 82.31%	1571, 57.50%	
Transfer method for patients	Emergency Medical Services	143, 18.74%	136, 4.98%	**0.000**
	Reaching hospital by oneself	620, 81.26%	2596, 95.02%	
Whether inter-hospital transfer was carried out	Yes	10,1.31%	259,9.48%	**0.000**
	No	753, 98.69%	2473, 90.52%	
**Medical history**				
Smoking	Present	222, 29.10%	741, 27.12%	0.281
	Absent	541, 70.90%	1991, 72.88%	
Drinking	Present	149, 21.85%	447, 17.48%	**0.009**
	Absent	533, 78.15%	2110, 82.52%	
Diabetes	Present	148, 19.40%	712, 26.06%	**0.000**
	Absent	615, 80.60%	2020, 73.94%	
Diabetes duration, y		7.58 ± 6.92	7.90 ± 6.26	0.586
Hypertension	Present	478, 62.65%	1774, 64.93%	0.243
	Absent	285,3 7.35%	958, 35.07%	
Hypertension duration, y		9.29 ± 8.82	8.13 ± 7.07	**0.003**
Hyperlipidemia	Present	69, 15.20%	428, 23.14%	**0.000**
	Absent	385, 84.80%	1422, 76.86%	
TIA or ischemic stroke	Present	329, 43.12%	1172, 42.90%	0.913
	Absent	434, 56.88%	1560, 57.10%	
Atrial fibrillation	Present	89, 11.66%	126, 4.61%	**0.000**
	Absent	674, 88.34%	2606, 95.39%	
Pre-onset mRS score		0.43 ± 1.01	0.38 ± 0.92	0.237
First symptom of AIS	Vomit	34, 4.46%	109, 3.99%	0.565
	Unconscious	77, 10.09%	107, 3.92%	**0.000**
	Paralysis	580, 76.02%	1998, 73.13%	0.110
	Diplopia	6, 0.79%	40, 1.46%	0.146
	Aphasia	93, 12.19%	152, 5.56%	**0.000**
	Dysarthria	276, 36.17%	844, 30.89%	**0.006**
	Drooping of angle of mouth	39, 5.11%	139, 5.09%	0.979
	Headache	8, 1.05%	78, 2.86%	**0.004**
	Paresthesia	100, 13.11%	306, 11.2%	0.473
	Vertigo	107, 14.02%	471, 17.24%	**0.034**
	Other symptoms	115, 15.07%	522, 19.11%	**0.011**
TOAST classification of stroke	LAA stroke	396, 51.90%	1204, 44.07%	**0.000**
	Cardiogenic stroke	126, 16.51%	220, 8.05%	
	SAO stroke	195, 25.56%	1139, 41.69%	
	SOE	17, 2.23%	51, 1.87%	
	Unexplained stroke	29, 3.80%	118, 4.32%	
Stroke severity	Mild stroke	380, 49.80%	1798, 65.81%	**0.000**
	Moderate stroke	283, 37.09%	787, 28.81%	
	Severe stroke	100, 13.11%	147, 5.38%	

ODT, onset-to-door time; AIS, acute ischemic stroke; LAA, Large-artery atherosclerosis; mRS, modified Rankin scale; SAO, Small artery occlusion; SOE, Stroke of other determined etiology; TOAST, Trial of Org 10,172 in Acute Stroke Treatment; y, year; TIA, Transient ischemic attack

Emergency Medical Services primarily involve the use of ambulances to transport patients

Medical insurance include social and commercial insurance

No smoking refers to not smoking at present or in the past

No drinking refers to not drinking at present or in the past

Mild, moderate, and severe strokes are defined as NHISS score of 0–4, 5–14 and 15–42, respectively

**Fig. 1:**
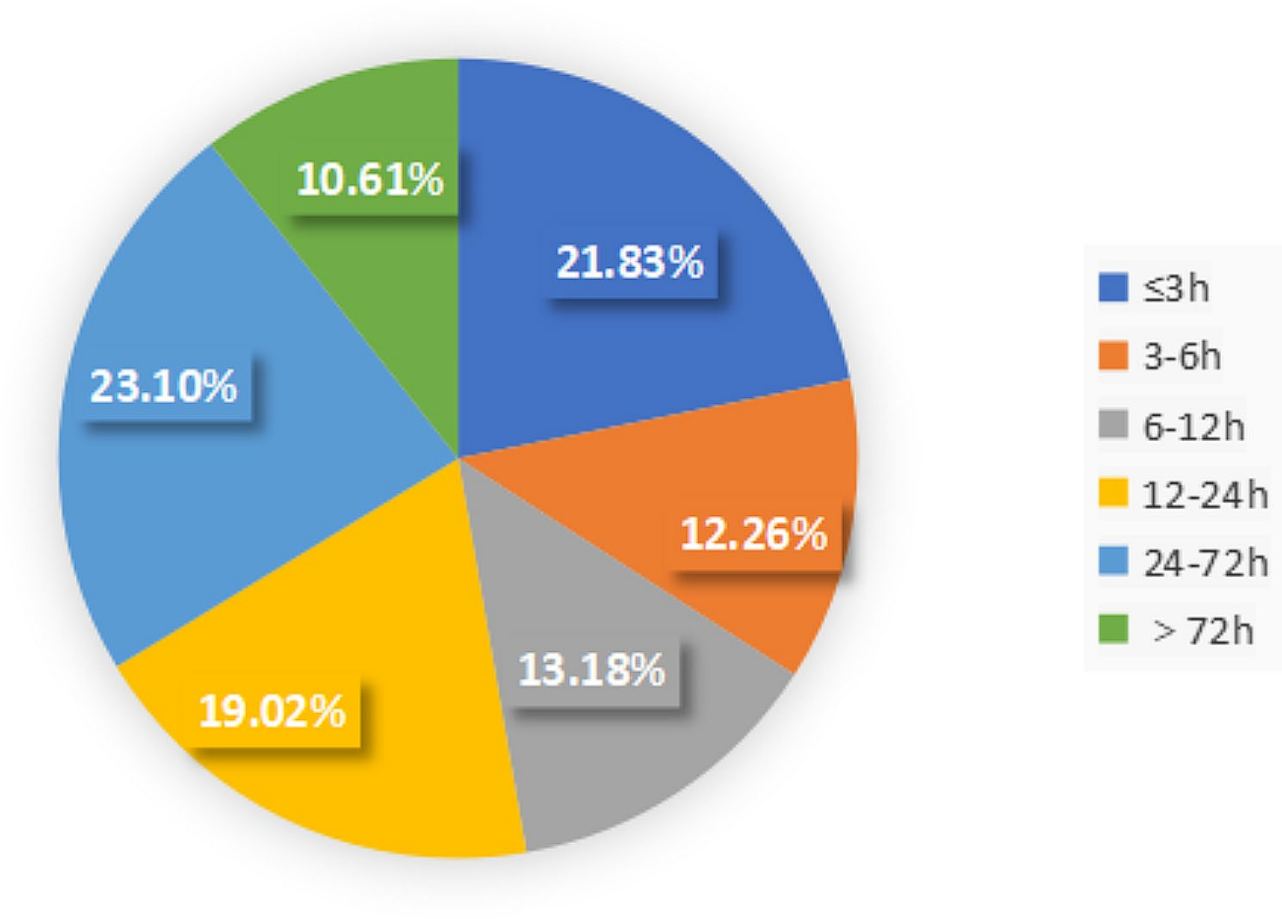
Distribution ratio of ODT in patients with acute ischemic stroke ODT: onset-to-door time

A total of 3495 patients with AIS were recruited, and the average is 1,698.88 min (range, 9–10,062 min), and the median is 883 min (Q1 = 181 min, Q3 = 2555 min). There were 763 patients (21.83%) with ODT ≤ 3 h and 2732 patients (78.17%) with ODT > 3 h. Specifically, the number of patients with ODT 3–6 h, 6–12 h, 12–24 h, 24–72 h, and > 72 h accounted for 12.26%, 13.18%, 19.02%, 23.10%, and 10.61% of all patients with AIS, respectively (Fig. 1). There were 2,317 patients (66.29%) with ODT ≤ 24 h and 1178 patients (33.71%) with ODT >24 h.

### Baseline characteristics and single-factor analysis of ODT in patients with acute ischemic stroke (Table 1)

Older patients (65.84 ± 11.52 years), lived in the city, had high educational qualifications, had a distance of > 20 km between onset location and the first-visit hospital, reached the hospital by ambulance, and had no inter-hospital transfer had higher ODT (*P* < 0.05).

There were significant differences in medical history (including current drinking, diabetes, hypertension duration, hyperlipidemia, and atrial fibrillation), the first symptom of AIS (including unconsciousness, aphasia, dysarthria, headache, and vertigo), TOAST classification of stroke, and stroke severity between ODT ≤ 3 h group and ODT > 3 h group (*P* < 0.05).

### Analysis of delayed ODT (> 3 h) with binary logistic regression analysis

Living in rural areas (OR: 1.478, 95% CI: 1.024–2.146) and existing interhospital transfer (OR: 7.479, 95% CI: 2.548–32.337) were risk factors for ODT (Table 2).

**Table 2:** Analysis of delayed onset-to-door time (> 3 h) with binary logistic regression analysis

	Adjusted Odds Ratio	95% confidence interval	*P*-value
Living in rural- areas	1.478	1.024–2.146	**0.038**
Distance between onset location and the first-visit hospital ≤ 20 km	0.355	0.236–0.530	**0.000**
Transportation- using EMS	0.346	0.216–0.555	**0.000**
Existing inter-hospital transfer	7.479	2.548–32.337	**0.001**
History of Atrial fibrillation	0.375	0.207–0.679	**0.001**
Moderate stroke	0.644	0.462–0.901	**0.010**
Severe stroke	0.506	0.285–0.908	**0.021**

Moderate and severe strokes are defined as NHISS score of 5–14 and 15–42, respectively

EMS, emergency medical service; The adjusted covariates include Gender, Age, Educational level, Drinking, Diabetes, Hyperlipidemia, First symptom of AIS, and TOAST classification of stroke

Distance between the onset location and first-visit hospital ≤ 20 km (OR: 0.355, 95% CI: 0.236–0.530), transportation of patients by EMSs (OR: 0.346, 95% CI: 0.216–0.555), history of atrial fibrillation (OR: 0.375, 95% CI: 0.207–0.679), moderate stroke (OR: 0.644, 95% CI: 0.462–0.901), and severe stroke (OR: 0.506, 95% CI: 0.285–0.908) were protective factors for ODT.

## Discussion

Our study demonstrated that only about one-fifth of patients with AIS could reach the hospital within 3 h of symptom onset, and the pre-hospital delay was significant. Some characteristics of pre-hospital delay are risk factors for ODT, such as living in rural areas and existing inter-hospital transfer; meanwhile, distance of ≤ 20 km between onset location and the first-visit hospital, transportation of patients by EMSs, and history of atrial fibrillation and moderate and severe stroke were protective factors for ODT.

### Comparison of ODT in China and developed countries

This study showed that the median of ODT was 852 min (range, 215–2459 min), and 21.83% of patients had ODT ≤ 3 h. A multicenter study in the United States showed that 21–40% of patients with AIS reach the hospital within 3 h of symptom onset [31]. In a 2006 study that included 62 subcenters in China that showed similar results, the median ODT was 15 h [13]. Our findings reveal that ODT has not shown much reduction after more than 10 years and is still 3–6 h longer than that in developed countries [30]. Furthermore, a study in 2012–2013 indicated that patients in China experienced more pre-hospital delays compared to those in the United States (1318 min vs. 644 min) [14]. There is a significant difference between ODTs in China and those in developed countries [9, 15, 32, 33].

The World Health Organization’s MONICA manual provides standardized guidelines for registering stroke events [26]. These guidelines ensure that stroke cases are consistently defined and registered, facilitating accurate comparisons across different populations and regions. The MONICA project has played a crucial role in standardizing the registration of acute stroke events, enabling uniform data collection and analysis [26]. Research has demonstrated that adherence to the MONICA criteria for stroke registration is essential for quality control and accurate event validation [34]. The protocols established by the MONICA project have been widely adopted in various studies for registering stroke events, highlighting the broad acceptance and utility of these guidelines [35]. The standardized approach to stroke event registration outlined in the MONICA manual is critical for ensuring the accuracy and consistency of data collected across diverse populations and periods.

Over time, the accuracy of patients’ and witnesses’ recollection of the onset time may diminish, posing a challenge in determining the ODT accurately. We have therefore implemented the following measures to address recall bias in our study design: utilizing standardized questionnaires and interview methods. Additionally, we observed that most patients experience an ODT of less than 1 day. Therefore, we argue that including all patients in the primary analysis, even those with an onset of illness exceeding 24 h, can offer a more comprehensive depiction of the actual situation.

### Residential area type and ODT

This study showed that living in rural areas was a risk factor for ODT. Only 8.18% of patients in rural areas in China reached the hospital within 3 h [36], while 45.8% of patients in urban areas reached the hospital within 3 h [37]. Compared with patients in urban areas, those in rural areas are typically older and have lower levels of education, poor housing conditions, and high poverty rates.

China’s economic and healthcare service development has been uneven. Medical and health services supply in China has obvious differences in spatial distribution [19], and the eastern region has the highest medical and health services supply level, followed by the western and central regions. Patients in some parts of China experienced pre-hospital delays owing to poor economic and sanitary conditions [3]. In rural areas, insufficient medical resources, low levels of medical care, and fewer medical staff members make it extremely difficult to meet the needs of patients with stroke. In addition, rural residents have limited access to medical knowledge about first aid; this often results in patients missing the optimal stroke treatment time [38]. The coverage and reimbursement rates of medical insurance in rural areas are lower than those in urban areas, and the frequency of rural patients visiting hospitals is also low [39, 40], which may also result in longer ODTs in rural areas compared with in urban areas.

### Distance between onset location and the first-visit hospital and ODT

Our study showed that the distance between the onset location and the initial hospital ≤ 20 km was associated with shorter ODT. Long distances are an important factor delaying patient transport. Improving transport efficiency is a solution that the EMS plays a crucial role in achieving. EMS most closely affects ODT [6]. When there is an optimal EMS, the median ODT can be reduced to 151 min, and the proportion of patients reaching the hospital within 3 h can be increased to 54% [6]. However, EMS usage adds to medical costs; therefore, EMS construction is not feasible in some areas. The awareness of patients regarding EMS usage is also relatively low, and the proportion of patients with AIS using it in China is extremely low, as shown in this study; our results are also consistent with the findings of Wang et al. [14].

### Stroke severity and ODT

Our findings showed that patients with moderate or severe stroke were more likely to reach the stroke center within 3 h after onset. Similar results have been reported by Iversen et al. [41]. Patients with moderate or severe stroke were more likely to arrive at the hospital promptly and receive reperfusion therapy. The more serious the stroke, the more it is likely to attract patients’ and bystanders’ attention; this was associated with a higher probability of using EMS. Our research suggests that patients with mild strokes often experience more delays, which can be attributed to several factors: (1) Atypical Symptoms: Mild strokes may manifest with subtle or non-specific symptoms that patients may not immediately recognize as indicative of a stroke. (2) Minimization of symptoms: Patients with mild strokes may diminish the severity of their symptoms or attribute them to other less serious conditions, delaying their decision to seek medical attention. (3) Fear or denial: Some patients may experience fear or denial about the possibility of experiencing a stroke. This psychological barrier can prevent them from promptly seeking medical care. (4) Neglect: Patients with mild strokes may perceive their symptoms as less urgent and may prioritize other obligations over seeking immediate medical attention. Thus, we emphasize the importance of concentrating on patients with mild stroke and the significance of timely referrals.

Bystanders are more likely to notice typical stroke symptoms such as limb weakness, speech disturbance, and walking difficulties [42]. It has been reported that living alone increases admission delay, and the recognition of symptoms by bystanders may shorten it [43]. The onset of symptoms can influence a patient’s decision-making. When dysarthria or decreased muscle strength were the first symptoms, the rate of hospital visits increased significantly within 4.5 h (*P* < 0.01) [6, 44, 45]. The more prominent the impact of the first symptom on daily living, the easier it is to attract the attention of patients and their families, the stronger the desire to seek medical attention, and the shorter the ODT. Only 53.8% of patients with posterior circulation stroke reach the hospital within 3 h, compared to 68.4% of patients with anterior circulation stroke [7]. Compared with dysphagia and limb weakness caused by posterior circulation stroke, posterior circulation stroke often presents with non-specific symptoms such as dizziness, vertigo, and nausea, which are easily attributed to poor rest, anxiety, and failure. Cryptogenic stroke is common in young people [46]. However, young patients often ignore the possibility of stroke onset, which leads to a pre-hospital delay. Patients often choose self-observation when stroke occurs and only visit the hospital if the symptoms persist or worsen because of the inability to identify stroke in an accurate and timely manner [13]. Recognizing symptoms of stroke is an independent factor associated with early arrival [47].

### Transportation of patients to hospital and ODT

Most patients in our study chose to go to the hospital by themselves, which increased the probability of inter-hospital transfer and caused pre-hospital delays. There are two main specific situations of interhospital transfer: (1) Patients who independently seek medical attention may arrive at a hospital without a stroke center, necessitating their transfer to one. (2) In our study, patients who utilized an ambulance were directly transported to a stroke center. However, interhospital transfers may occur for these patients if the initial hospital cannot administer mechanical thrombectomy treatment. Only one in eight patients with stroke in China arrived at the hospital via EMS [48], compared to 59.6% in the DASH II study [49]. Our study showed that patients who visited the hospital via EMSs (ambulances) had shorter ODTs. Moreover, many studies have demonstrated a reduction in pre-hospital delays via EMS [50, 51]. The 2019 AHA/ASA guidelines indicate that patients with stroke who use EMS arrive at the emergency department earlier, and more eligible patients receive IVT [27]. A stroke emergency map (an intelligent EMS that can guide ambulances to transport patients more effectively) in China has effectively shortened the ODT and improved the thrombolysis rate [52].

### Atrial fibrillation (AF) and ODT

Our study demonstrated that patients with AF are not prone to pre-hospital delays, given that strokes resulting from atrial fibrillation tend to be more severe [53, 54]. Cardiac stroke typically occurs abruptly with evident symptoms. Patients often experience obvious discomfort, which helps in raising the alert faster, causing them to seek medical attention in time. The multivariate regression analysis revealed that AF was an independent factor associated with early arrival [47]. AF and a history of coronary artery disease accelerated the presentation to the hospital [13]; sudden onset of symptoms, loss of consciousness, recognition of symptoms as stroke, and feelings of fear and panic were associated with hospital arrival within 3 h.

### The impact of the COVID-19 pandemic on patients with stroke

The COVID-19 pandemic has markedly impacted patients with stroke, affecting different aspects of stroke care. Studies have demonstrated a decrease in hospital admissions for transient ischemic attacks and mild to moderate stroke during the COVID-19 era [55, 56]. Additionally, the pandemic has disrupted the chain of acute stroke care, resulting in potential risks such as decreased thrombectomy rates [57, 58] and modifications in the acute stroke care pathway [59]. Furthermore, the pandemic has caused a delay in patients with AIS seeking treatment at stroke centers [4]. Both pre- and post-hospital delays have been considerably prolonged, and the number of patients receiving intravenous thrombolysis treatment has decreased [60].

Moreover, a higher occurrence of severe strokes and an increased in-hospital mortality rate have been observed during the COVID-19 pandemic [61]. The pandemic has also raised concerns about the collateral damage on stroke emergency services, as well as the necessity to reorganize stroke networks in order to provide optimal care while mitigating the risk of transmission [55]. In conclusion, the COVID-19 pandemic has had a multifaceted impact on patients with stroke, affecting various aspects of stroke care, including hospital admissions, acute stroke care pathways, delayed presentation, and treatment.

### Limitations

The stroke population recruited in each subcenter of this study had certain regional characteristics; therefore, generalization of the research conclusions was affected to some extent. This study lacks detailed information on imaging, timing of EMS notification, and adjustment for socioeconomic factors. These limitations may have specific implications for interpreting and inferring research results: (1) Lack of imaging information: Inability to accurately assess disease severity and progression. (2) Lack of information on EMS notification time: Inability to determine the timeliness of patient medical assistance and the absence of a reference for optimizing emergency response systems. (3) Failure to adjust for socioeconomic factors: Socioeconomic status may influence patients’ healthcare-seeking behavior. Neglecting socioeconomic factors may introduce bias, potentially leading to an overestimation or underestimation of the impact of certain factors. Lastly, the COVID-19 pandemic presented significant challenges for this study. Lockdown restrictions and safety concerns led to limited data collection, resulting in a smaller sample size.

## Conclusions

Pre-hospital delays for patients with AIS are a serious medical and social issue that needs immediate attention. The majority (approximately four in five) of patients with AIS fail to reach the hospital within a 3-h prime time for stroke treatment, leaving much room for improvement in this regard. Reasonable distribution of hospitals that provide treatments to residents staying nearby, minimizing interhospital transfers, paying special attention to patients with moderate or severe stroke, and encouraging patients to reach the hospital by ambulance are recommended measures that can help reduce pre-hospital delays. The findings should be interpreted considering the constraints imposed by the COVID-19 pandemic. Future longitudinal studies could investigate the lasting effects of the pandemic on the research topic.

## Data Availability

The datasets used and/or analyzed during this study are available from the corresponding author upon reasonable request.

## Abbreviations

AF: Atrial fibrillation
AIS: Acute ischemic stroke
EMS: Emergency medical services
IVT: Intravenous thrombolysis
mRS: Modified Rankin scale
NHISS: National Institutes of Health Stroke Scales
ODT: Onset-to-door time
TOAST: Trial of Org 10,172 in acute stroke treatment
